# Factors Associated With Engagement When Using Social Media to Teach Physicians Point-of-Care Ultrasound During Off-Hours: Observational Study

**DOI:** 10.2196/94540

**Published:** 2026-09-29

**Authors:** Katelyn A Seward, Gagandeep Kaur, Paul J Han, Michelle C Higginson, Samantha R Spierling Bagsic, Bruce J Kimura

**Affiliations:** 1 Scripps Mercy Hospital San Diego, CA United States; 2 Scripps Health San Diego, CA United States

**Keywords:** point-of-care ultrasound, graduate medical education, engagement, internet, residency

## Abstract

**Background:**

Although social media is often viewed by residents and could be used to reinforce teaching points, there is little data on methods that improve engagement in learning medical topics while socially perusing this medium.

**Objective:**

We observed how the timing of posted questions, correctly answering questions, and providing supportive comments affected resident engagement with point-of-care ultrasound clips viewed electively on a private social media account.

**Methods:**

Of 60 medical residents, 35 followed an Instagram account that posted ultrasound video clips with questions during the academic year. Engagement was defined as the percentage of questions answered among the total number of clips viewed, calculated for each posted story (story engagement, E(s)) and each resident (resident engagement, E(r)). E(s) was tested for an association with (1) weekend vs weekday posts, (2) answering questions correctly vs incorrectly, and (3) supportive responses from faculty vs no feedback.

**Results:**

Of 16 posts, 120 questions were answered from 428 clips viewed by 35 residents, resulting in an E(s) of 28% (range 15%-59%) across posts and a median E(r) of 19% (IQR 0%-39%) across residents; 71% (n=25) of residents engaged with at least 1 post. E(s) was higher during weekdays vs weekends (30% vs 21%; *P*=.007) and correlated to answering correctly vs incorrectly (*r*=0.6; *P*<.001). A supportive comment resulted in a lower percentage of answering the next post, compared to no feedback (30% vs 71%; *P*=.02).

**Conclusions:**

Social media engagement was higher when questions were answered correctly but, surprisingly, lower when posts were made on weekends and immediately after receiving a supportive comment.

## Introduction

In younger generations, social media is often used as an escape from academic or work-related stress and may be addictive [1]. However, immensely popular apps, such as Instagram, Twitter or X, and TikTok, are gaining traction in the medical community as a tool for disseminating medical news; fostering collaboration and professional development; and, more recently, enhancing medical education [2-5]. When surveyed, many physicians who use social media view it as an efficient and effective tool to remain up to date and deliver quality patient care [2]. The incorporation of virtual learning into graduate medical education was augmented by the COVID-19 pandemic, as many programs adapted by using social media [6-9]. Online visual media has gained popularity among medical trainees, largely in the form of concise infographics to disseminate research and facilitate education [10-13]. The short, focused format of social media posts could be incorporated into a traditional didactic curriculum as a low-cost, interactive tool to reinforce key teaching points, so-called “pearls,” during a clinical rotation. Various medical education training programs have begun to explore medical education using text and image-based posts. Surveys of participating medical learners reflect a positive view of social media as an educational tool, with both perceived and measurable benefits for learners who interact with the posts [14-16].

The aforementioned studies involved unblinded participation by willing residents who accessed social media with the intent to learn. However, when social media is accessed for its intended social purposes, it is unclear whether users would welcome educational posts. Maintaining user engagement would be indispensable for teaching on social media, but it could be difficult, especially if scrolling through a work-related topic is considered an unexpected intrusion. Few data exist to guide medical educators in crafting posts that will draw engagement from learners as they review their daily social media feeds. Therefore, to elucidate learner behaviors, we present groundwork observations on whether the weekend timing of posts, answering questions correctly, and receiving supportive comments resulted in higher engagement by residents electively accessing an educational point-of-care ultrasound (POCUS) account on Instagram (Meta Platforms).

## Methods

### Overview

The study was conducted within the graduate medical education program at Scripps Mercy Hospital, which developed training in POCUS through the cardiovascular limited ultrasound examination (CLUE) program [17,18]. The CLUE program was incorporated into the 3-year internal medicine residency curriculum in 2006. We conducted a retrospective review of data during the 2023 to 2024 academic year from an Instagram account followed by internal medicine and transitional year medical residents that focused on CLUE, a topic in which no resident had prior training. POCUS education requires visual content and repeated opportunities to reinforce key concepts and image recognition to optimize learning. Visual-based social media platforms such as Instagram are well suited to complement the core POCUS curriculum. In 2016, the program created a private CLUE Instagram account, initially used to make program announcements, but soon included teaching points and video clips with questions, evolving into an extracurricular learning opportunity. The account’s existence was simply mentioned at resident orientation during description of their whole POCUS curriculum. Residents chose to follow the CLUE Instagram account on a purely voluntary basis, often under their own online pseudonym. Residents were not coerced to view or engage with questions and had no expectation of subsequent testing. To assess this medium as a teaching method, the Instagram account was queried retrospectively to assess responses and engagement, starting with the new academic year. On this platform, we posted a “story,” a brief transitory video made by the account owner that the follower can view privately for only 24 hours, and later a corresponding “post,” a permanent video automatically placed in the feed of all followers and archived in the account. Instagram user account names were anonymized by assigning each user a unique identification number by the chief resident for analysis.

Typically, a 2- to 3-second video loop that had been saved within the smartphone app of the POCUS device was transferred to Instagram with a multiple-choice question superimposed on the side of the clip using a feature in Instagram (Figure 1). The video, along with its question, was first posted as a story and subsequently as a post containing the answer and explanation. The chief resident largely determined this frequency based upon her and her colleagues’ familiarity with Instagram and the residency program to maintain novelty and interest. The CLUE program director composed all the stories, posted them at his convenience, and did not view the resident responses. The question was constructed to relate to the CLUE curriculum and included simple interpretation of images, clinical correlations, and pearls or pitfalls. The timing of the posted story, between weekdays and weekends or during day hours (defined as 8 AM to 5 PM) or morning or evening hours (defined as before 8 AM or after 5 PM), was not controlled. After posting the story, the chief resident recorded which residents had viewed it, attempted the question, and answered correctly. With this methodology, only the user and account owner know if a specific user answered correctly. Instagram considers a story “viewed” once the user opens the clip. The platform tallies the total number of users who “viewed” the clip within the platform, a number displayed for 48 hours that only the account owner can view. These proprietary industry marketing algorithms could not be altered.

**Figure 1:**
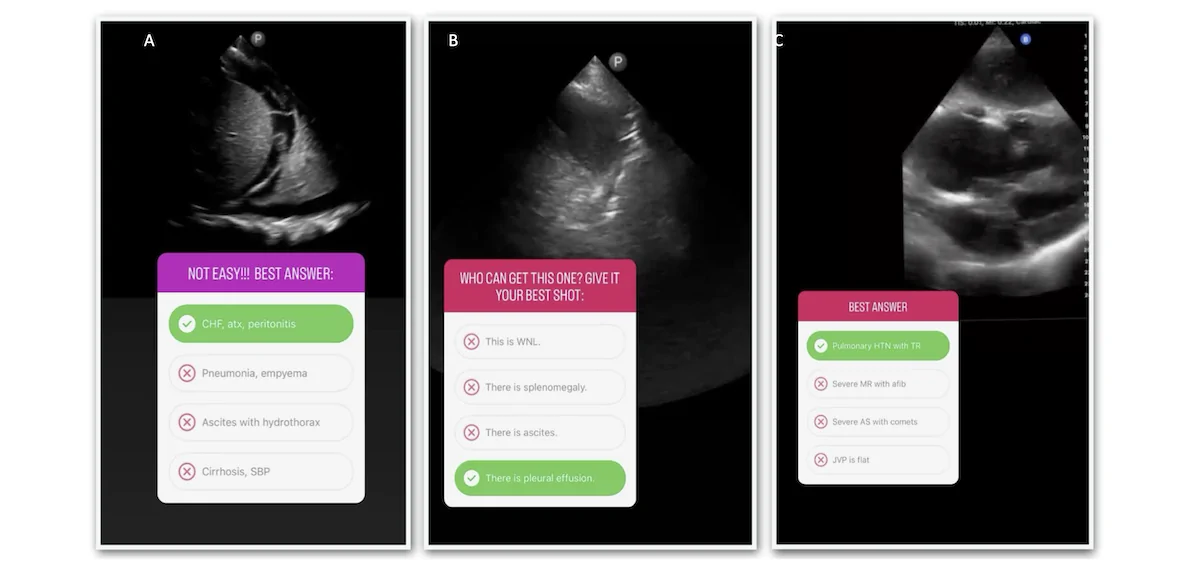
Examples of stories posted on the cardiovascular limited ultrasound examination (CLUE) Instagram account (still images from point-of-care ultrasound videos) with associated multiple-choice questions. Calculated engagement (story engagement, E(s)) for each story, representing the percentage of residents who both viewed the story and answered the associated question: (A) E(s)=33%, (B) E(s)=15%, and (C) E(s)=59%.

Story engagement, E(s), was calculated as follows: E(s)=100%×(number of residents who answered the question/number of residents who viewed the story).

E(s) represented how many residents viewed and answered the question, a topic-centric measure of popularity of that particular story. Resident engagement, E(r), was calculated as follows: E(r)=100%×(number of questions answered by resident/number of stories viewed by resident).

E(r) was a resident-centric measure of a resident’s participation over all the questions viewed by that resident. The percentage of correct answers was calculated for each story. Within the following week, a corresponding post explaining each answer shown in the video was published for all followers to see. Importantly, unlike the story, the post video could be permanently archived by category (eg, heart images, lung images, and carotid images) in the account for users to access as an ongoing library, which could be reviewed in its entirety by users in the future to fill in any developing gaps in learning.

To test whether an interactive response helped resident engagement, the chief resident provided a brief “supportive comment,” regardless of whether the question was answered correctly or not. For each story, residents who answered correctly each privately received the same reaffirming comment (ie, “Great job, knew you could do it!”), and those who answered incorrectly each privately received the same encouraging comment (ie, “Good try, better luck next time!”). These story comments and replies, unlike responses to posts, are not seen by other users and were private communications between the Instagram account owner and follower. During the first 5 months of the academic year, approximately one-half of the residents received brief, private, supportive comments, while the other half received no comments. During the second 5 months, a crossover occurred: residents who had previously received comments stopped receiving them, while residents who had not previously received any comments began receiving supportive comments.

The total duration of the project, in terms of data reviewed, had been predetermined as approximately 1 year, beginning at the start of the academic year and ending when approximately equal times were spent in the commenting vs no commenting periods by the residents. Therefore, by 10 months, all anonymized data that had been recorded were analyzed by a dedicated statistician and reviewed by POCUS faculty to inform changes for the following academic year.

### Ethical Considerations

The retrospective review of anonymized educational data was presented to the Scripps Health Institutional Review Board as a teaching performance improvement project and was granted an exemption from review, with waiver of the requirement for informed consent. All data were anonymized.

### Statistical Analysis

Story and resident engagement values, E(s) and E(r), respectively, are reported as medians with IQRs. E(s) was tested for an association with (1) timing of weekday vs weekend posts, (2) answering questions correctly vs incorrectly, and (3) supportive comments from faculty vs no comments. Because of the small convenience sample and violation of normality assumptions, nonparametric tests were used. Differences between groups were tested using the Mann-Whitney *U* test, correlation was tested with the Spearman rho rank coefficient, and a *P* value <.05 was considered significant.

## Results

Of 60 residents, 35 (58%) willingly followed the private CLUE Instagram account. All 35 residents had a previous account on Instagram, and 17 (49%) were female. Over 10 months, a total of 16 stories were created. Assuming a potential total of 560 story views (35 participants×16 stories), 428 (76%) story views were recorded. A total of 120 questions were answered across 428 clips viewed, resulting in an E(s) of 28% (range 15%-59%) and a median E(r) of 19% (IQR 0%-39%). Of the 35 residents, 25 (71%) answered at least 1 story. The study did not define a threshold for “sufficient” resident engagement or coerce any engagement, as supported by the fact that 10 residents had E(r)=0: 3 residents never viewed any stories, and 7 residents never answered any questions despite viewing stories.

Regarding the timing of the 16 stories, 9 were released on weekdays and 7 on weekends. Story engagement was found to be significantly higher during weekdays vs weekends, with E(s) of 30% (22%-59%) vs 21% (15%-29%), respectively, (*P*=.007). When subcategorized by time of the day, the 5 weekday evening posts had, on average, the most story engagement, E(s)=35%, followed by the 4 weekday 8 AM to 5 PM posts, E(s)=30%; the 3 weekend morning or evening posts, E(s)=24%; and the 4 weekend 8 AM to 5 PM posts, E(s)=21%.

Of the 120 resident answers, 64 (53%) were answered correctly. Spearman correlation analysis revealed a moderate positive association between the percentage of questions answered correctly and E(r) (ρ=0.6; *P*<.001; Figure 2). Three outliers answered 100% of questions correctly but rarely engaged, suggesting selectivity in when they responded.

**Figure 2:**
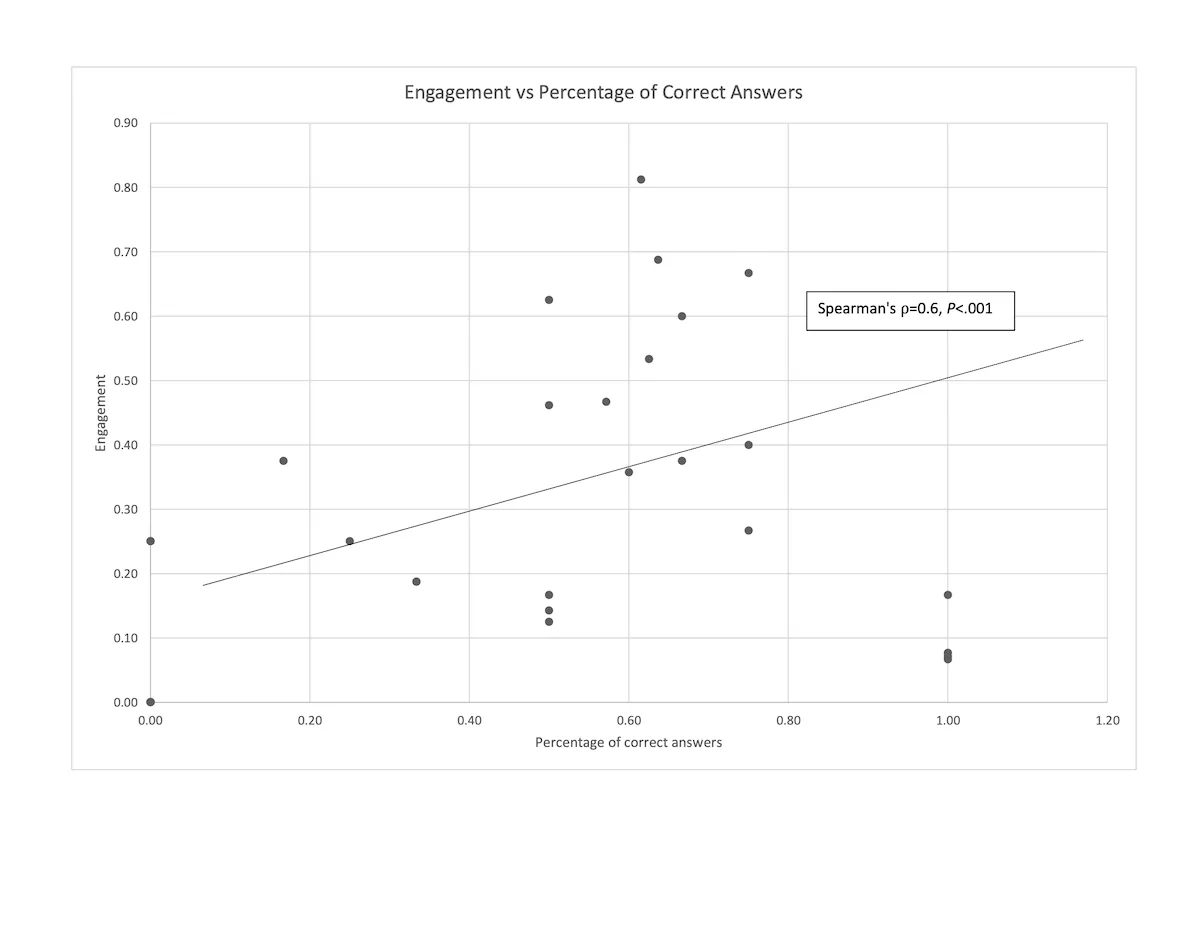
Engagement vs percentage of correct answers. Scatter plot showing the correlation between resident engagement (E(r)) and the percentage of correct answers over the study period (16 posts over 10 months), demonstrating a positive correlation (*P*<.001). Each point represents 1 resident.

Regardless of whether the answer was correct, supportive comments were sent in response, and 72 instances had no feedback provided. The effect of receipt of a supportive comment vs no comment on resident engagement revealed median E(r)=12% (IQR 0%-41%) vs E(r)=16% (IQR 0%-40%), respectively, (*P*=.28). Residents who received a supportive comment were found to have a significantly lower E(s) with the next posted story compared to residents who received no comment (22/60, 36% vs 35/60, 58%; *P*=.02). To explore the effect of receiving comments, story engagement before and after the crossover point at 5 months was analyzed to examine trends. Overall, E(s) was 30% just before the crossover point (31% for the initial “no comment” group vs 24% for the initial “comment” group) and decreased to 27% after the second 5-month period (19% for the initial “no comment” group vs 25% for the initial “comment” group).

## Discussion

### Principal Findings

Over the course of an entire medical residency, the current study found that 58% (35/60) of residents voluntarily followed an Instagram POCUS account for 10 months; 76% (428/560) of posted stories were viewed, and 28% (120/428) of the viewed stories generated active engagement. These findings uniquely document the proportion of residents willing to voluntarily engage in an educational message during review of social posts. While using social media to teach resident physicians, we observed that story engagement was higher when residents answered questions correctly and when posts were made on weekdays rather than weekends. Surprisingly, supportive comments from faculty were associated with an immediate decrease in story engagement and had no significant overall effect on resident engagement. This study reveals factors associated with elective resident engagement on a popular social media site and its use of a specific topic, POCUS, with which internal medicine residents were unfamiliar and may seek extracurricular teaching. These observations may serve as pilot data or groundwork to optimize this novel form of interactive medical education.

This investigation leveraged the addictive behaviors of social media sites, such as checking stories on Instagram, to disseminate brief medical teaching points to residents during their off-hours or downtime. The current retrospective analysis of purely elective Instagram use is unlike prior investigations in which participants knew that their behavior was being observed and is also unlike other online teaching methods, such as educational websites, podcasts, or YouTube videos, where the user voluntarily and passively listens to material for self-learning or as a part of a curriculum. Instead, this study “intruded” into the resident’s social feed with an unannounced, brief learning opportunity, such as reinforcing a “pearl” from the day’s lecture or rounds, during a time they had relegated to personal social activity. Therefore, the magnitude and factors involved in engagement are essential to understand, as it was an on-the-spot decision by the learner on whether to participate while swiping through stories. The study uniquely provides rare “real-world” behavioral observations of this decision to engage through its retrospective crossover design, assessing the so-called Hawthorne effect, which occurs when participants alter their behavior upon becoming cognizant of being observed [19].

Prior studies have found that short social media videos can be useful for teaching subjects such as radiology and anatomy across various stages of medical education and may improve short-term academic performance [15]. However, the tendency for passive learning remains a common and largely unexplored area of concern. Most studies on medical education through social media measure passive engagement by the number of followers, likes, comments, or hashtag use, with varying amounts of learner participation, and often measure perceived benefit of the learner as an outcome [14,20-23]. In the current study, pairing each posted POCUS video clip with a question offered a unique way to measure more active rather than passive learner engagement. The unexpected finding of higher story engagement during busy weekdays than on weekends suggests that time off may be perceived as an opportunity for rejuvenation and avoidance of work-related topics, even when engagement requires only a brief time commitment. This finding also aligns with concerns that the rapid access to educational content on social media can be a “double-edged sword,” as learners may use social media during personal downtime and may perceive educational content appearing in their feeds negatively [24].

Posting “pearls” or “take home points” to a resident’s social media feed allows residency programs to emphasize key concepts or provide much-needed amid the vast amount of educational material learners encounter. Because story engagement was higher when residents were able to answer questions correctly, targeting a difficulty level that keeps a wide range of learners both engaged and challenged will likely require tactful planning, potentially individualized through algorithms based on learner profiles. Surprisingly, receipt of a supportive comment did not improve subsequent story engagement, as the lack of anonymity to the faculty may have resulted in answering only those stories in which the correct answer was known. This finding is contrary to the results of other studies assessing social media use in undergraduate medical education and medical students that suggests social media positively supports communication between learners and educators [14,25]. Potentially, this points towards unforeseen differences in undergraduate vs graduate medical education, in terms of user age, concerns of anonymity, and class size. This dilemma also illustrates another challenge of integrating social media into medical training, which is the lack of privacy, as social media platforms are inherently public [23] and blend the social and professional aspects of life. Future considerations could include peer-level or automated comments.

The current study has several limitations. First, the definitions of engagement likely underestimated residents’ interest and actual learning, as residents may have learned from the video clip, read the question, chosen not to answer, and instead awaited the subsequent annotated post or reviewed the archived library. Therefore, the sustained 19% voluntary story engagement over months may be good for any social media user interaction that requires an actual response from the viewer. Interestingly, this percentage may represent underlying personality traits specific to engaged residents that relate to their willingness to participate, despite answering incorrectly. Second, this study could not elucidate reasons why a POCUS story was not viewed or was “viewed” but not read (ie, quickly swiped over). It is possible that unviewed or swiped-through clips reflected residents’ lack of time to interact with the POCUS story while it was active. Assessing time spent on educational vs social posts would require intrusive monitoring and could introduce additional biases. Although viewing a story does not guarantee an actual educational interaction, it must be emphasized that, on this medium, learning cannot occur without first viewing the clips. Therefore, understanding the factors that affect the subsequent engagement is fundamental. This study lays the groundwork on how to post, a critical step in elective use, so that future studies can evaluate optimal learning outcomes. Third, as a single-center retrospective study, we could not control for confounding factors, such as the diverse nature and trends during residency and social influences on engagement; hence, we enrolled chief residents to help design this project. Finally, proprietary algorithms that promote posts, inherent to social media, could not be controlled or entirely accounted for, but we do not believe our findings regarding posting and user behavior are specific to the platform used in this study.

### Conclusions

Social media is a wide-reaching and powerful platform that can be used as an inexpensive method to reinforce teaching points in medical training. Interjecting a brief medical lesson from that week’s conference or daily rounds into a resident’s social media feed could provide unexpected learning opportunities during off-hours. We found that posts were generally welcomed and that engagement was higher when questions were answered correctly but, surprisingly, lower when posts were made on weekends or immediately after a supportive comment was received. These findings should be considered when using social media as a supplemental, elective teaching tool in medical education.

## Data Availability

The datasets analyzed during the current study are anonymized but not publicly available due to the searchable nature of social media and to ensure user privacy. The anonymized datasets are available from the corresponding author on reasonable request.

## Abbreviations

CLUE: cardiovascular limited ultrasound examination
E(r): resident engagement
E(s): story engagement
POCUS: point-of-care ultrasound
